# Valorization of Blue Crab (*Callinectes sapidus*) By-Products into Antioxidant Protein Hydrolysates for Nutraceutical Applications

**DOI:** 10.3390/ani15202952

**Published:** 2025-10-11

**Authors:** Rosaria Arena, Simona Manuguerra, Michelle Marchan Gonzalez, Elena Petrosillo, Davide Lanzoni, Clément Poulain, Frédéric Debeaufort, Carlotta Giromini, Nicola Francesca, Concetta Maria Messina, Andrea Santulli

**Affiliations:** 1 Laboratory of Marine Biochemistry and Ecotoxicology, Department of Earth and Marine Sciences DiSTeM, University of Palermo, Via Barlotta 4, 91100 Trapani, Italy; rosaria.arena@unipa.it (R.A.); simona.manuguerra@unipa.it (S.M.); michellemagriet.marchangonzalez@unipa.it (M.M.G.); andrea.santulli@unipa.it (A.S.); 2 Department of Veterinary Medicine and Animal Science, University of Milan, Via dell’Università 6, 26900 Lodi, Italy; elena.petrosillo@unimi.it (E.P.); davide.lanzoni@unimi.it (D.L.); carlotta.giromini@unimi.it (C.G.); 3 Department of BioEngineering, IUT-Dijon-Auxerre, University Burgundy Europe, BP 17867, 21000 Dijon, France; clement.poulain@ube.fr (C.P.); frederic.debeaufort@ube.fr (F.D.); 4 Joint Unit A02.102 PAM-PAPC—Physical Chemistry of Food and Wine Laboratory, Université Bourgogne Europe-Institut AgroDijon-INRAé, 1 Esplanade Erasme, 21000 Dijon, France; 5 Institute for Food, Nutrition and Health, University of Reading, Reading RG6 5EU, UK; 6 Department of Agricultural, Food and Forest Sciences (SAAF), University of Palermo, Viale delle Scienze, Bldg. 5, 90128 Palermo, Italy; nicola.francesca@unipa.it

**Keywords:** blue crab by-products, protein hydrolysates, antioxidant activity, HS-68 cell line

## Abstract

**Simple Summary:**

The Atlantic blue crab is an invasive species that has spread in the Mediterranean Sea, causing serious impacts on marine biodiversity, fisheries, and tourism. Although its meat is highly valued, most of the crab is discarded during processing, generating large amounts of waste. Developing strategies to use these by-products is essential to reduce environmental impact and create new opportunities for sustainable blue growth. In this study, we have examined the nutritional value and bioactive properties of blue crab (*Callinectes sapidus*) processing by-products, with a particular focus on protein hydrolysates. These were tested for their ability to act as antioxidants using both chemical methods and in vitro assays on human skin fibroblast cell line. The results showed that hydrolysates have strong antioxidant activity and can protect cells from oxidative damage. This demonstrates that waste from blue crabs can be converted into valuable ingredients for food, animal feed, and nutraceutical applications, providing a sustainable way to manage an invasive species while adding economic value.

**Abstract:**

The Atlantic blue crab (*Callinectes sapidus*) is an opportunistic invasive species in the Mediterranean that is negatively affecting biodiversity, fisheries, and tourism. In Italy, it is appreciated for its good meat quality, but the processing yield is low (21.87 ± 2.38%), generating a significant amount of by-products (72.45 ± 4.08%), which are underutilized. Valorizing this biomass is in line with circular economy principles and can improve both environmental and economic sustainability. This study aimed to valorize Atlantic blue crab by-products (BCBP), producing protein hydrolysates and assessing their in vitro bioactivities, in order to plan applications in animal food and related sectors. BCBP hydrolysates were obtained by enzymatic hydrolysis using Alcalase and Protamex enzymes. The treatment with Alcalase resulted in a higher degree of hydrolysis (DH = 23% in 205 min) compared to Protamex (DH = 14% in 175 min). Antioxidant activity of the hydrolisates was evaluated through DPPH, ABTS, reducing power and FRAP assays, as well as in vitro test in fibroblasts (HS-68). At 10 mg/mL, hydrolysates from both enzymes exhibited the maximum radical scavenging activity in DPPH and ABTS assays. In HS-68 cells, 0.5 mg/mL hydrolysates protected against H_2_O_2_-induced oxidative stress, showing a cell viability comparable to cells treated with 0.5 mM N-acetyl cysteine (NAC), as an antioxidant. Statistical analyses were performed using one-way ANOVA followed by Student–Newman–Keuls (SNK) or Games–Howell post hoc tests, with significance set at *p* < 0.05. Overall, both enzymes efficiently hydrolyzed BCBP proteins, generating hydrolysates with significant antioxidant activity and cytoprotective effects. These results demonstrate the potential to produce high-quality bioactive compounds from BCBPs, suitable for food, nutraceutical, and health applications. Scaling up this valorization process represents a viable strategy to improve sustainability and add economic value to the management of this invasive species, turning a problem in a resource.

## 1. Introduction

The Atlantic blue crab *Callinectes sapidus* (Rathbun, 1896) (Brachyura: Portunidae), native to the western Atlantic from Uruguay to Nova Scotia, was accidentally introduced into the Mediterranean in the early 20th century, likely via ballast water, and deliberately into the Aegean Sea in the 1930s. Since then, it has expanded across the Mediterranean, Black Sea, and European Atlantic coasts [1,2,3]. Its opportunistic feeding, high reproductive output, habitat flexibility, and competitive behavior have made it one of the 100 most invasive marine species in the Mediterranean, where it strongly affects native biodiversity and human activities such as fisheries, aquaculture, and coastal economies [1,4,5,6].

Despite its negative impacts, *C. sapidus* could be considered as a potential economic resource due to its high nutritional value [7]. Atlantic blue crab meat contains approximately 14–19% proteins and is rich in polyunsaturated fatty acids, such as arachidonic acid (ARA; C20:4 n6), eicosapentaenoic acid (EPA; 20:5 n3), and docosahexaenoic acid (DHA 22:6 n3), which are known for their beneficial effects on human health [7].

However, the edible yield is very low (10–15%), and the processing generates considerable amounts of waste, primarily in the form of shells. These by-products are often considered as low-value materials, yet they are a promising source of high-value compounds such as chitin, proteins, polyunsaturated fatty acids, minerals, and carotenoids [7,8]. Special attention should nevertheless be paid to the content of heavy metals, particularly cadmium (Cd) and arsenic (As) in crab shells, especially in areas affected by industrial pollution, to ensure the safety of potential food, nutraceutical, and feed applications [9,10].

In recent years, the valorization of these crustacean by-products has attracted increasing interest within the blue and circular economy, offering opportunities to both reduce waste and develop value-added products for industry applications [7,11]. To date, most studies on *C. sapidus* residues have focused on chitin recovery. In contrast, the protein fraction, which represents around 30% of the biomass, remains comparatively underexplored [7,12,13].

This is a significant gap, given that proteins from marine organisms, including fish and crustaceans, are recognized as a promising source of bioactive peptides with antihypertensive, antithrombotic, immunomodulatory, and antioxidant properties [14,15,16,17]. Such bioactivities make them highly attractive for nutraceutical, pharmaceutical, cosmetic, and functional food applications [15,18,19,20,21].

In order to obtain bioactive peptides from marine by-products, a variety of extraction methods may be employed, including acid-alkaline hydrolysis, enzymatic hydrolysis, and fermentation. Among these, enzymatic hydrolysis is considered the most suitable for applications in the food and pharmaceutical sectors, as it avoids the use of toxic chemicals and organic solvents while ensuring high specificity and bioactivity of the resulting peptides [17,22]. Despite this, the potential of enzymatically derived peptides from *C. sapidus* by-products remains largely unexplored, representing a critical knowledge gap, considering both the ecological threat posed by the species and the urgent need for sustainable valorization strategies [7].

This study aimed to address this gap by testing the hypothesis that blue crab by-products can be effectively utilized as a novel source of bioactive peptides with antioxidant properties. Specifically, we (i) characterized the biochemical and nutritional composition of the Atlantic blue crab by-products (BCBPs) to evaluate their potential as a source of high-value nutrients and (ii) produced protein hydrolysates via enzymatic hydrolysis and assessed their antioxidant activity through chemical and in vitro assays. This integrated approach provides insight into the potential applications of these compounds in the pharmaceutical and nutraceutical sectors and also contributes to sustainable resource utilization. By valorizing marine by-products that would otherwise be considered low-value waste, it promotes circular economy strategies and supports the development of environmentally friendly, high-value product.

## 2. Materials and Methods

### 2.1. Raw Material

The Atlantic blue crab by-products (BCBPs) utilized in this study were obtained from a processing facility in Palermo, Sicily, and collected during the summer season. The material, consisting of shells and legs, was subjected to a standard industrial processing step of boiling at 100 °C for 20 min. The claws, legs and body meat were manually separated, and the weight of the recovered meat was recorded to calculate the processing yield (Y) according to the following equation:

Y = M/WW − 100(1)

where M is the weight of the removed meat and WW is the total wet weight of the crab [23].

The BCBPs were stored in cold containers, transported to the laboratory, and frozen at −20 °C. Prior to analysis they were rinsed with water, dried in a ventilated thermostat at 37 °C for 24 h, subsequently ground to a particle size of approximately 2 mm, and divided into three portions of 250 g each, which were vacuum-packed in plastic bags and stored at −80 °C. Each portion was treated as an independent sample for all subsequent analyses.

### 2.2. Evaluation of Proximate Composition of Blue Crab By-Products

The proximate composition was analyzed in three replicates. Moisture and ash contents were determined according to the AOAC official method [24], while total lipids were extracted using the Folch method [25]. Crude protein content was calculated from total nitrogen determined by the Kjeldahl method [26], using a conversion factor of 6.25. The value was corrected to account for nitrogen associated with chitin, according to the following formula [27]:

(2) $$\mathrm{Protein}\;\mathrm{content}\;\left(\%\;\mathrm{dw}\right)=\;\left(\mathrm{Total}\;N-\mathrm{Chitinous}\;N\right)\times 6.25$$

Chitinous N was determined as follows [27]:

(3) $$\mathrm{Chitinous}\;N\;\left(\%\;\mathrm{dw}\right)=\frac{\mathrm{Chitin}\;\mathrm{content}\times 6.89}{100}$$

where 6.89% is the theoretical nitrogen factor corresponding to completely acetylated chitin [27,28,29].

Chitin was extracted from BCBP following three steps. First, the sample was demineralized with 1 M HCl (1:10 *w*/*v*) at room temperature for 24 h to remove minerals. The residue was then deproteinized with 1 M NaOH (1:10 *w*/*v*) at 90 °C for 2 h, followed by repeated washing with distilled water to neutrality. Subsequently, the material was decolorized with 1% sodium hypochlorite solution for 30 min, and dried at 60 °C for 4 h in a forced-air oven according to Bolat et al. [30]. After drying, the chitin yield was calculated and expressed as a percentage of total BCBP.

### 2.3. Enzymatic Hydrolysis of Blue Crab By-Products

BCBP (10 g) was resuspended in distilled water (1:10 *w*/*v*) in a 250 mL vessel with continuous stirring and pH monitoring according to Messina et al. [31]. Two bacterial proteases were used: Alcalase (≥2.4 U/g, Merck KGaA, Darmstadt, Germany) and Protamex (>1.5 AU-N/g, Merck KGaA, Darmstadt, Germany). Enzymes were applied at an enzyme/substrate (E/S) ratio of 3% under their respective optimal conditions, according to the manufacturers, 53 °C and a pH of 9.0 and 8.5 for Alcalase and Protamex, respectively. The pH was maintained by the addition of 5 N NaOH.

The degree of hydrolysis (DH%) of each enzyme was determined every 15 min over a 265 min period, using the equation reported by Dumay et al. [32] and Messina et al. [15]. The enzymatic activity was stopped by heating the samples at 90 °C for 5 min. The hydrolysates were centrifuged at 7142× *g* force for 15 min at 4 °C using an Eppendorf Centrifuge 5430 R (Eppendorf, Hamburg, Germany). The resulting supernatants were lyophilized, stored at 4 °C. All hydrolyses were performed in triplicate independent experiments.

### 2.4. Protein Hydrolysates Characterization and Assessment of Antioxidant Activity

#### 2.4.1. Sodium Dodecyl Sulphate-Polyacrylamide Gel Electrophoresis (SDS-PAGE)

Protein molecular weight was evaluated by SDS-PAGE for both BCBP and protein hydrolysates. Protein concentration in all samples was determined by the Lowry method [33], using bovine serum albumin (BSA; Merck KGaA, Darmstadt, Germany) as a standard.

Aliquots containing 60 µg of protein in a final volume of 20 µL were mixed with Laemmli buffer (Merck KGaA, Darmstadt, Germany), denatured at 90 °C for 5 min, and loaded onto a 4–15% gradient polyacrylamide minigel (Bio-Rad, Hercules, CA, USA). Electrophoresis was performed at 20 mA for about 2 h using a Mini-PROTEAN Tetra Cell electrophoresis system (Bio-Rad, Hercules, CA, USA) with Tris-glycine running buffer (pH 8.3). A protein standard mix (8–220 kDa) was run in parallel. After electrophoresis, the gel was stained with Coomassie Blue (Bio-Rad, Hercules, CA, USA), and the image was acquired using a Chemi Doc XRS (Bio-Rad, Hercules, CA, USA) and analyzed with Image Lab 4.1 software (Bio-Rad, Hercules, CA, USA) [15]. Three independent gels were run.

#### 2.4.2. DPPH Radical Scavenging Activity

The antioxidant activity of protein hydrolysates was evaluated using the DPPH radical scavenging assay [15]. Serial dilutions of the hydrolysate stock solution (30 mg/mL in water) were prepared to final concentrations of 0.5, 1.0, 2.5, 5.0, and 10.0 mg/mL. Aliquots of 0.4 mL of each dilution were mixed with 1.6 mL of 100 μM DPPH in 96% ethanol. The mixtures were incubated at room temperature for 30 min in the dark, and the absorbance (A) was then measured at 517 nm using a Multiskan microplate reader (Thermo Fisher Scientific™, Waltham, MA, USA). Gallic acid (Merck KGaA, Darmstadt, Germany) was used as a positive control.

All measurements were performed in six replicates.

The percentage of radical scavenging activity was calculated according to the following equation:

(4) $$I\%=1-\left(\frac{A_{\mathrm{sample}}}{A_{\mathrm{blank}}}\right)\times 100$$

where A_sample_ is the absorbance of the sample and A_blank_ is the absorbance of the blank.

#### 2.4.3. ABTS Radical Scavenging Activity

The antioxidant activity of the protein hydrolysates was evaluated using the ABTS (2′-azinobis-(3-ethylbenzothiazoline-6-sulfonic acid)) assay, according to the Re et al. method [34] with modifications described by Lanzoni et al. [35]. Each hydrolysate was tested at five concentrations (0.5, 1, 2.5, 5, and 10 mg/mL) by diluting the stock solutions (30 mg/mL) in water.

A 7 mM ABTS solution and a 140 mM potassium persulfate solution were prepared in distilled water (Merck KGaA, Darmstadt, Germany). These two solutions were then mixed to generate the ABTS radical cation (ABTS•+), which was allowed to develop in the dark at room temperature for 16 h. Before use, the ABTS•+ solution was diluted with ethanol until an absorbance of 0.70 ± 0.02 at 734 nm was achieved. For the antioxidant activity measurement, 20 µL of each sample was added to 2.0 mL of the ABTS•+ working solution (A734 nm = 0.700 ± 0.020), incubated for 6 min at room temperature in the dark, and then the absorbance (A) was measured at 734 nm using a Multiskan microplate reader (Thermo Fisher Scientific™, Waltham, MA, USA).

All measurements were performed in six replicates.

The percentage of inhibition was determined using the following equation:

(5) $$I\%=\left(\frac{A_{\mathrm{sample}}}{A_{\mathrm{blank}}}\right)\times 100$$

where A_sample_ is the absorbance of the sample and A_blank_ is the absorbance of the blank.

#### 2.4.4. Ferric-Reducing Antioxidant Power (FRAP) Assay

The Ferric-Reducing Antioxidant Power (FRAP) assay was performed on the hydrolysates following the method described by Abdelaleem and Elbassiony [36], with some modifications as detailed in Lanzoni et al. [35]. Each hydrolysate was tested at five different concentrations (0.5, 1, 2.5, 5, and 10 mg/mL), prepared by diluting the stock solutions in water (30 mg/mL). The FRAP reagent was prepared by mixing three solutions: 300 mM acetate buffer (pH 3.6), obtained by dissolving 2.69 g of sodium acetate trihydrate in 16 mL of glacial acetic acid and bringing the final volume to 1.0 L with distilled water; 10 mM TPTZ (2,4,6-tripyridyl-s-triazine) dissolved in 40 mM HCl; and 20 mM ferric chloride hexahydrate in distilled water. For the assay, 300 µL of FRAP reagent was mixed with 10 µL of sample, and the reaction mixture was incubated in the dark at room temperature for 20 min. Absorbance was then measured at 595 nm using a Multiskan microplate reader (Thermo Fisher Scientific™, Waltham, MA, USA). All measurements were performed in six replicates.

The standard was Acid Ascorbic. Results were expressed as µmol Ascorbic Acid Equivalents (AAE)/L.

#### 2.4.5. Reducing Power

The reducing power of the BCBP hydrolysates was assessed using the spectrophotometric iron reduction method [37,38]. Hydrolysates were tested at five concentrations (0.5, 1, 2.5, 5, and 10 mg/mL). For the assay, 0.2 M phosphate buffer (pH 6.6) and 1% (*w*/*v*) potassium ferricyanide [K_3_Fe(CN)_6_] were mixed with the hydrolysate solution in a 1:1:1 (*v*/*v*/*v*) ratio and incubated at 50 °C for 20 min. An equal volume of 1% (*w*/*v*) trichloroacetic acid was then added, and the mixture was centrifuged at 3000 rpm for 10 min. From the upper layer, distilled water and 0.1% (*w*/*v*) FeC1_3_ were added in a 1:1:2 (*v*/*v*/*v*) ratio. Absorbance was measured at 700 nm using a Multiskan microplate reader (Thermo Fisher Scientific™, Waltham, MA, USA). All measurements were performed in six replicates. Gallic acid (GAE) was used as a positive control (all reagents were obtained from Merck KGaA, Darmstadt, Germany).

#### 2.4.6. Cell Culture

Human skin fibroblasts (HS-68; ECACC n. 89051701) were grown as a monolayer in Dulbecco’s Modified Eagle’s Medium (DMEM) supplemented with 10% fetal bovine serum (FBS), 2 mM glutamine, and 100 µg/mL penicillin–streptomycin. Cells were incubated at 37 °C with 5% CO_2_, under sterile conditions (all reagents were obtained from Merck KGaA, Darmstadt, Germany).

#### 2.4.7. Protective Effects of BCBP Hydrolysates

Confluent cells were trypsinized and seeded in a 96-well plate (Nunc, Darmstadt, Germany) at 7.5 × 10^4^ cells/well and incubated for 24 h. Preliminary experiments were conducted to determine the non-cytotoxic range of BCBP hydrolysates obtained from Alcalase and Protamex. Cells were treated with hydrolysate concentrations ranging from 0.5 to 50 mg/mL (dissolved in sterile distilled water and filtered through a 0.22 µm Millipore membrane, Millex^®^ Merck Millipore, Darmstadt, Germany) for 24 h. Cell viability was assessed using the 3-(4,5-dimethyl-2-yl)-2,5-diphenyltetrazolium bromide (MTT; Merck KGaA Darmstadt, Germany) assay [39], as described by Messina et al. [40]. Concentrations ≥10 mg/mL significantly reduced cellular viability, whereas 0.5 mg/mL did not affect cellular viability. Therefore, 0.5 mg/mL was selected for the subsequent oxidative stress protection assay. This allowed evaluation of potential protective effects against hydrogen peroxide-induced stress without interfering with cellular viability.

For the oxidative stress assay, HS-68 cells were treated with 0.5 mg/mL of BCBP hydrolysates from Alcalase and Protamex for 24 h. Control cells were maintained only with culture medium, while a positive control group was treated with synthetic antioxidant 0.5 mM N-acetyl cysteine (NAC) (Merck KGaA Darmstadt, Germany). After 24 h, all treated samples (except the control) were exposed to hydrogen peroxide (H_2_O_2_; 10 µM) for 2 h at 37 °C, according to a standardized protocol [38,41,42]. Cell viability was then measured using the MTT assay [39] and expressed as a percentage of viable cells relative to the control. Each experiment of viability was carried out in five replicates.

### 2.5. Statistical Analysis

Data were analyzed using SPSS for Windows^®^ (version 20.0, SPSS Inc., Chicago, IL, USA). Results are expressed as mean ± standard deviation. Homogeneity of variance was confirmed by the Levene test. One-way analysis of variance (ANOVA) was applied, followed by Student–Newman–Keuls (SNK) or Games–Howell post hoc tests for multiple comparisons. The significance level was 95% (*p* < 0.05) in all cases.

## 3. Results and Discussion

### 3.1. Yield and Proximate Composition

Atlantic blue crab meat provides a high content of essential nutrients, including proteins, omega-3 fatty acids, vitamins, and minerals, contributing to its nutritional value [7]; however, its low yield results in considerable waste [43]. The main problem in crab meat production is the process of meat removal [44]. In the present study, where the meat was removed manually, the meat yield was 21.87 ± 2.38 (Table 1), as reported by Yomar-Hattori et al. [23]. Consequently, a large portion of the crab (72.45 ± 4.08%; Table 1) is discarded as processing by-products.

These by-products have been shown to be rich in chitin, protein, minerals, fatty acids, and carotenoids [7].

One of the most critical initial steps in planning the utilization of marine by-product biomasses within a circular economy framework is the evaluation of their nutritional composition and bioactive potential, particularly for applications in the food, feed, and nutraceutical sectors [22].

The proximate composition of BCBP (dw) is reported in Table 2. A high ash content was found (35.38 ± 0.30), although this value was lower than that reported by Antunes-Valcareggi et al. [12] (56.2 ± 1.2%). Such elevated ash levels hinder the direct use of these by-products as fish meal in aquaculture, as ash contents above 12% can impair fish growth by reducing the digestibility of other dietary ingredients [45].

A lipid content of 2.41 ± 0.08% was observed, in accordance with Antunes-Valcareggi et al. [12], but higher than the 0.41 ± 0.01% reported by Félix-Valenzuela et al. [13]. Data on the biochemical composition of blue crab by-products remains limited in the literature. Similarly low lipid contents have been reported in other crab by-products, such as the southern king crab (*Lithodes santolla*) (0.5%) [45], green crab shells (*Carcinus maenas*) (0.37–0.67%) [46], and hard shell (2.41 ± 0.30%) and soft shell crabs (1.50 ± 0.7%) of *Portunus sanguinolentus* [47]. Overall, lipid content in crustacean by-products has been reported to range from 0.2 to 17% [48,49] with variability depending on the species, the geographical location of the fishery, and the nature of the by-products [48].

As shown in Table 2, BCBP is characterized by a high protein content (31.21 ± 1.12%), representing the second most abundant nutrient. This value is in line with previous findings, such as those reported by Antunes-Valcareggi et al. [12] (33 ± 1%) and Félix-Valenzuela et al. [13] (33.8 ± 2.3%), and is comparable to protein contents observed in other crab by-products [50].

Proteins and their derivatives, including hydrolysates, peptides, and amino acids, constitute a significant proportion of crustacean by-products [7,51]. Typically, a high protein content (25–50%) is reported in crustacean by-products [48,52], making their valorization particularly relevant. Numerous studies have focused on the identification and characterization of bioactive peptides derived from crustacean by-products, given their potential applications in the prevention of various pathologies, the development of functional foods, and the production of edible films [20,48,53,54].

In addition to protein, chitin represents a critical component of crustacean by-products [55]. In the present study, the chitin content was 10.57 ± 0.66% (Table 2), consistent with previous findings on the same species [7,56,57,58]. A similar value has been reported in other crab species [27,50,59], although chitin levels can vary depending on the specific body parts analyzed [59].

### 3.2. Degree of Hydrolysis

In this study, protein hydrolysates were obtained by enzymatic hydrolysis using commercially available proteases according to a standardized protocol [15] previously applied to other crustacean species [15]. This environmentally friendly method allows the deproteinization of crustacean by-products, generating protein hydrolysates with high nutritional value [12,60,61].

**Figure 1:**
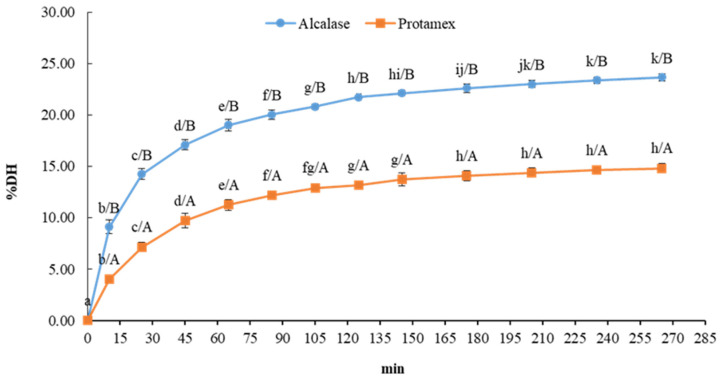
Degree of hydrolysis (% DH) for blue crab by-products (BCBP) treated with Alcalase and Protamex over time. Data are presented as mean ± standard deviation (*n* = 3). Lowercase letters (a, b, c…) indicate significant differences between different time points for the same enzyme (*p* < 0.05). Uppercase letters (A; B; …) indicate significant differences between the two different enzymes at the same time point (*p* < 0.05).

Hydrolysis of the blue crab by-products was performed using two enzymes, Alcalase and Protamex, with Alcalase demonstrating significantly higher efficiency. As shown in Figure 1, Alcalase achieved a higher degree of hydrolysis (DH%), reaching a maximum of 23% in 205 min, compared to Protamex, which peaked at 14% in 175 min. The superior performance of Alcalase, an enzyme derived from *Bacillus licheniformis*, suggests it has a higher specific affinity for the protein substrate [12].

Research conducted on other marine by-products (such as *Callinectes ornatus* and *Portunus sanguinolentus*) has shown a higher efficiency of the enzyme Alcalase compared to Protamex [12,62,63,64,65]. Thus, Alcalase appears to be one of the most effective enzymes in the preparation of fish and crustacean protein hydrolysates due to its high proteolytic activity on different protein sources [62,64,65,66]. The superior performance of Alcalase is likely due to its broad substrate specificity and higher affinity for BCBP proteins, confirming its suitability for scalable production of crustacean protein hydrolysates. Similarly, the antioxidant capacity measured here is in agreement with findings on crab and shrimp by-products (e.g., *Lithodes santolla* and *Carcinus maenas*), confirming that crustacean wastes are a promising source of bioactive peptides [45,46].

Protein hydrolysates are particularly valuable ingredients due to their richness in bioactive peptides, with antioxidant and antimicrobial properties. These properties allow them to be incorporated into food products as natural preservatives, thereby enhancing shelf life while concomitantly providing health-promoting benefits [11]. This renders them essential components in functional foods and nutraceutical applications [64,67,68].

The degree of hydrolysis (DH%) is a key parameter in the characterization of protein hydrolysates. The findings of the present study demonstrate DH% above 10%, indicating a high peptide yield. This level significantly enhances protein solubility, which is essential for the efficient downstream processing of hydrolysates, including their recovery and purification, and for their successful incorporation into various food additives and industrial applications [64,67]. Furthermore, the resulting protein hydrolysates exhibit strong potential as high-value food ingredients, as their effectiveness within complex food matrices is largely determined by their functional properties, with solubility remaining the main determining factor. Indeed, the manifestation of other texture-defining attributes, including surface-active functions (e.g., foaming and emulsifying) and gel formation, presupposes that the protein is adequately solubilized within the food system [11]. Protein hydrolysates are of considerable interest not only for their role in improving food quality but also for their bioactivity, which is highly valued in the pharmaceutical and seafood industries [64,65]. However, it must be acknowledged that the primary focus of the study was strictly on the biochemical composition and antioxidant potential of the BCBP hydrolysates. Consequently, other functional and technologically relevant properties, including solubility, emulsifying, foaming, and gelling capacities, were not characterized in this work.

Moreover, this approach aligns with circular economy principles, allowing the valorization of processing waste into high-value ingredients, potentially reducing environmental impact while creating economic opportunities.

### 3.3. Characterization and Bioactivity Evaluation of Protein Hydrolysate

#### 3.3.1. SDS-PAGE

**Figure 2:**
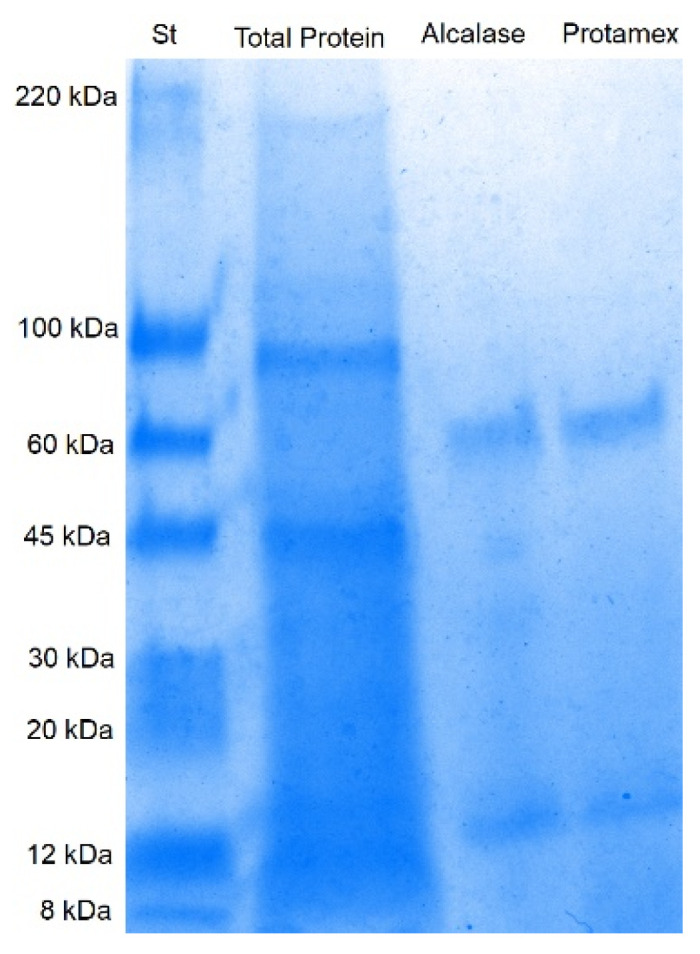
SDS-PAGE of the total proteins and protein hydrolysates, obtained with the enzymes Alcalase and Protamex from BCBP powder at the end of the enzymatic process.

The electrophoretic profile of the protein hydrolysates (Figure 2) revealed a marked reduction in the intensity of high molecular weight bands compared to the total extracted proteins. Both Alcalase and Protamex exhibited strong proteolytic activity, as evidenced by the generation of lower molecular weight peptides around 65 kDa and 12 kDa. These observations confirm the efficiency of the enzymatic hydrolysis process in the breakdown of proteins into smaller peptide fragments.

Overall, these findings underscore the effectiveness of both enzymes in valorizing marine by-products, with Alcalase often considered as the most efficient protease to produce fish and crustacean protein hydrolysates due to its broad substrate specificity and high catalytic activity [12]. A study on shrimp by-products reported that Alcalase hydrolysis produced protein hydrolysates with significant antioxidant activity, as evidenced by SDS-PAGE analysis showing a broad distribution of peptide sizes [69]. Similarly, research on stone fish (*Actinopyga lecanora*) revealed that Alcalase digestion produced antioxidant proteolysates. SDS-PAGE profiles demonstrated the formation of peptides of varying molecular weights [70].

#### 3.3.2. Antioxidant Activity

**Figure 3:**
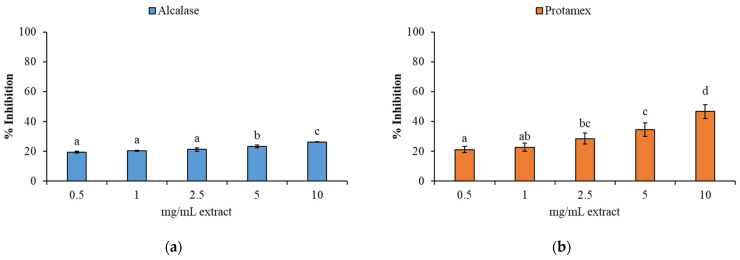
1,1-diphenyl-2-picryhydrazyl (DPPH) radical inhibition (%) of BCBP protein hydrolysates at different concentrations (0.5, 1, 2.5, 5, and 10 mg/mL extract) obtained by hydrolysis with (**a**) Alcalase and (**b**) Protamex. Different letters (a, b, c…) indicate significant differences between the different concentrations of extract tested for each enzyme (*p* < 0.05). Data are presented as mean ± standard deviation (*n* = 6).

The protein hydrolysates demonstrated the ability to scavenge DPPH radicals, with results expressed as % inhibition (Figure 3a,b). At concentrations of 0.5 and 1 mg/mL, no statistically significant differences were observed between the hydrolysate obtained with Alcalase (Figure 3a) and Protamex (Figure 3b). Both enzymes exhibited their maximum radical scavenging activity at the highest tested concentration of 10 mg/mL (Figure 3a,b).

The DPPH assay confirmed that BCBP protein hydrolysates exhibit significant antioxidant properties. They act as potent electron donors capable of neutralizing free radicals and effectively terminating the radical chain reaction by converting them into stable molecules. These results are in accordance with previous studies on by-products from other crustaceans, including crab [64,71] and shrimp by-products [15,44,62,72].

The BCBP protein hydrolysate demonstrated the ability to inhibit substrate oxidation, indicating its potential to reduce oxidative stress [64,73,74,75,76]. Both enzymes utilized in this study showed comparable inhibitory potency at the lowest concentrations (Figure 3a,b), supporting their suitability for producing bioactive antioxidant peptides.

**Figure 4:**
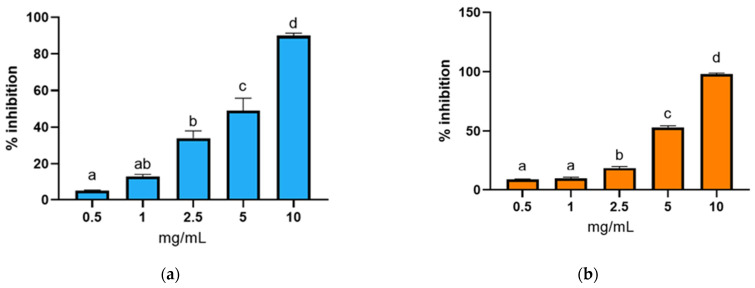
ABTS test inhibition (%) of BCBP protein hydrolysates at different concentrations (0.5, 1, 2.5, 5, and 10 mg/mL extract) obtained by hydrolysis with (**a**) Alcalase and (**b**) Protamex. Different letters (a, b, c…) indicate significant differences between the different concentrations of extract tested for each enzyme (*p* < 0.05). Data are presented as mean ± standard deviation (*n* = 6).

The ABTS assay (Figure 4a,b) corroborated these results, showing that hydrolysates obtained with Alcalase and Protamex achieved their maximum radical inhibition at the highest tested concentration (10 mg/mL). This finding is consistent with the trends that were observed in the DPPH assay.

DPPH and ABTS are synthetic radicals used to assess antioxidant activity because they react with antioxidant compounds via hydrogen atom transfer (HAT) and single-electron transfer (SET) mechanisms [77]. As Martínez-Montaño [78] observed in fish protein hydrolysates, the results of the DPPH and ABTS assays suggest that the predominant antioxidant mechanism is hydrogen atom transfer (HAT). This is likely due to the presence of numerous hydroxyl groups (–OH) and aromatic structures that facilitate the transfer of hydrogen atoms by antioxidant groups in the protein hydrolysates [77]. The similar trends observed in both the DPPH and ABTS assays further support the hypothesis that this behavior is primarily attributable to the HAT mechanism.

**Figure 5:**
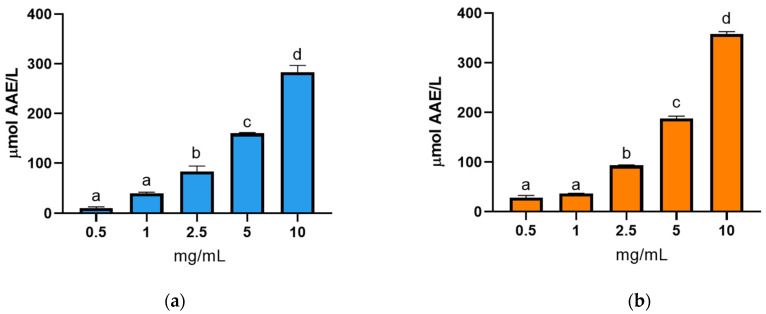
FRAP test (expressed as mg ascorbic acid equivalents (AAE) mg/100 g) of BCBP protein hydrolysates at different concentrations (0.5, 1, 2.5, 5, and 10 mg/mL extract) obtained by hydrolysis with (**a**) Alcalase and (**b**) Protamex. Different letters (a, b, c…) indicate significant differences between the different concentrations of extract tested for each enzyme (*p* < 0.05). Data are presented as mean ± standard deviation (*n* = 6).

**Figure 6:**
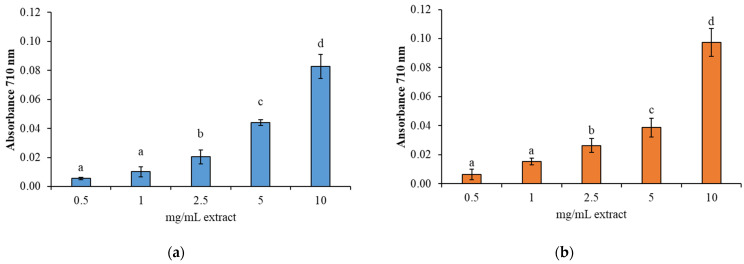
Reducing power of BCBP hydrolysates at different concentrations (0.5, 1, 2.5, 5, and 10 mg/mL extract) obtained by hydrolysis with (**a**) Alcalase and (**b**) Protamex. Different letters (a, b, c…) indicate significant differences between concentrations for each enzyme (*p* < 0.05). Data are presented as mean ± standard deviation (*n* = 6).

The antioxidant activity of BCBP hydrolysates was also evaluated using two different assays: the reducing power and FRAP assay (Figure 5 and Figure 6). These methods evaluate the capacity of antioxidant compounds to donate electrons or hydrogen atoms, thereby reflecting their ability to reduce ferric ions (Fe^3+^) to ferrous ions (Fe^2+^). Studies have shown that there is a direct correlation between antioxidant activities and the reducing power of certain bioactive compounds [71,79,80].

A dose-dependent increase in antioxidant activity was observed for both enzymatic treatments, indicating that higher hydrolysate concentrations significantly enhanced the ferric ion-reducing potential. This finding indicates that the peptides resulting from hydrolysis possess significant electron-donating properties. The results of the reducing power and FRAP assays showed consistent and parallel trends, thereby reinforcing the robustness of the findings. These observations are in alignment with the results of the DPPH (Figure 3) and ABTS (Figure 4) radical scavenging assays, which also demonstrated a concentration-dependent antioxidant response. The comparable behavior exhibited by all four assays suggests that the antioxidant mechanism is likely dominated by hydrogen atom or electron transfer processes, potentially due to the presence of hydroxyl groups and aromatic residues in the hydrolysates [71,77,78,79].

The observed antioxidant effects are likely related to the amino acid composition of the hydrolysates. Specific amino acids, such as histidine, tyrosine, methionine, and cysteine, are known to enhance radical scavenging activity through hydrogen donation, electron transfer, or metal ion chelation [81]. Our results are consistent with previous studies on crustacean by-products, in which the presence of these amino acids was associated with enhanced radical scavenging activity [81]. Overall, these findings suggest that BCBP hydrolysates could be used as bioactive ingredients in functional food and nutraceuticals to counteract oxidative stress.

#### 3.3.3. Antioxidant Capacity of Hydrolyzed Fractions In Vitro, at the Cellular Level

**Figure 7:**
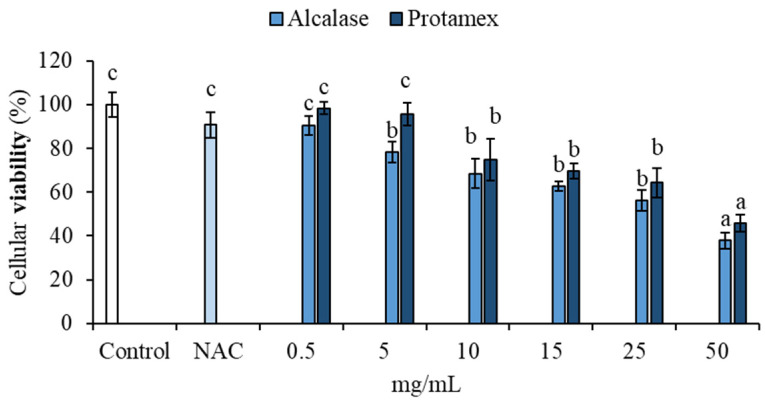
Cytotoxicity effect of BCBP hydrolysates at different concentrations (0.5, 5, 10, 15, 25, and 50 mg/mL) in HS68 measured by MTT assay. Control: untreated cells; NAC: cells pretreated with the synthetic antioxidant N-acetyl cysteine (0.5 mM); 0.5–50 mg/mL: cells treated with different concentrations of BCBP hydrolysates for 24 h. Data are presented as mean ± standard deviation (*n* = 5). Different lowercase letters (a, b, c…) indicate significant differences between concentrations for each enzyme (*p* < 0.05).

The HS68 cell line was treated with BCBP hydrolysates obtained from the enzymes Alcalase and Protamex at concentrations of 0.5, 5, 10, 15, 25 and 50 mg/mL for 24 h. A significant dose-dependent decrease in cell viability was observed at concentrations of 10, 15, 25, and 50 mg/mL of BCBP hydrolysates produced by both enzymes, compared to the control group (Figure 7). These findings are consistent with Camargo et al. [64], who reported a similar dose-dependent reduction in the viability of pre-osteoblast cells when treated with *C. ornatus* protein hydrolysates produced using the same enzymes [64].

The lowest tested concentration (0.5 mg/mL) did not affect cell viability for either enzyme (Figure 7) and also exhibited comparable antioxidant activity (Figure 3 and Figure 4).

**Figure 8:**
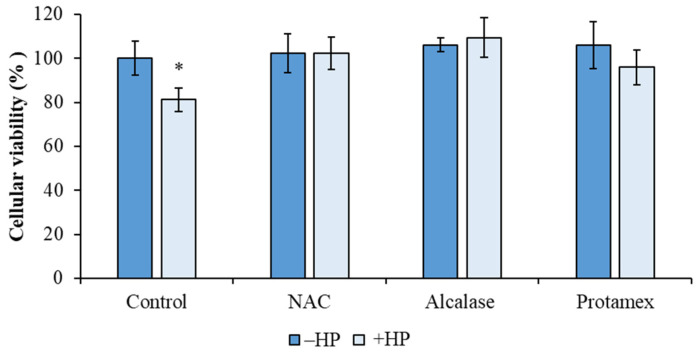
Effect of BCBP hydrolysates (0.5 mg/mL) obtained from Alcalase and Protomex on HS68 fibroblast cells exposed to oxidative stress induced by H_2_O_2_ (+HP). Control: untreated cells; NAC: cells treated with N-acetyl cysteine (0.5 mM). Alcalase: cells pretreated with BCBP hydrolysates obtained by hydrolysis with Alcalase. Protamex: cells pretreated with BCBP hydrolysates obtained by hydrolysis with Protamex. Bars represent mean ± standard deviation (*n* = 5). Statistical differences (*p* < 0.05) vs. Control (−HP) are indicated by “*”.

Therefore, 0.5 mg/mL was selected for further evaluation of the protective effects against oxidative stress induced by hydrogen peroxide. As shown in Figure 8, HS68 cells pretreated with 0.5 mg/mL BCBP hydrolysates from either enzyme and then exposed to hydrogen peroxide exhibited higher levels of viability than untreated stressed cells (+HP). These protective levels were comparable to cells treated with the synthetic antioxidant N-acetyl cysteine (NAC).

This study demonstrates that BCBP hydrolysates exert a protective antioxidant effect against oxidative damage. Notably, no previous studies have reported on the antioxidant activity of hydrolyzed blue crab fractions in human fibroblast cells, highlighting a significant gap in the current literature. The high bioactivity observed in this study is strongly supported by prior research on marine-derived protein hydrolysates, which are well known to exhibit potent antioxidant properties through free radical scavenging and chelating pro-oxidative metal ions [15,82].

Our results suggest that the protection of HS-68 fibroblasts from H_2_O_2_-induced oxidative stress involves both direct and indirect antioxidant effects. The hydrolysates may directly scavenge reactive oxygen species (ROS) and chelate pro-oxidative metal ions, thereby reducing oxidative damage [83]. Furthermore, studies on marine-derived protein hydrolysates suggest that these peptides can activate endogenous cellular antioxidant defense [84,85,86]. For instance, Sun et al. [84] demonstrated that antioxidant peptides derived from marine protein hydrolysates activate the Keap1-Nrf2 pathway, leading to the upregulation of detoxifying and antioxidant enzymes such as superoxide dismutase (SOD), catalase (CAT), and glutathione peroxidase (GPx). Similarly, Wang et al. [85] reported that collagen peptides from *Harpadon nehereus* bones improved antioxidant abilities by regulating this pathway, while Xu et al. [86] confirmed that marine-derived peptides can activate Nrf2-mediated expression of phase II detoxifying/antioxidant enzymes, thereby enhancing the cellular antioxidant defense system. These findings support the hypothesis that the protective effect of BCBP hydrolysates is rooted in this dual mechanism of action, which significantly strengthens their translational value for industrial applications. Further research is needed to elucidate these pathways and determine whether specific peptide fractions contribute preferentially to the observed protective effects. The evidence of both direct ROS scavenging and indirect activation of endogenous antioxidant enhances the potential of these hydrolysates for wide-ranging industrial applications [85]. In the food sector, they could be employed as natural preservatives to improve product stability and extend shelf-life [11]. An especially innovative approach involves their incorporation into chitosan gelatin biopolymer films for active food packaging, where they could provide antioxidant protection against oxidative degradation, a strategy already explored for other crustacean protein hydrolysates [20]. Beyond the food sector, hydrolysates are of considerable interest for their bioactivity, which is highly valued in the nutraceutical and pharmaceutical industries. In addition to food and nutraceutical uses, the findings highlight potential applications of blue crab hydrolysates in the animal feed industry [11]. Their high protein content and antioxidant activity make them suitable candidates as functional ingredients in feed formulations, particularly in aquaculture, where sustainable alternative protein sources are increasingly sought. The inclusion of these hydrolysates could contribute to improving animal health while also reducing reliance on conventional fishmeal [11,87]. However, further studies are required to assess their digestibility, safety, and in vivo efficacy across different animal models, to fully validate their potential for industrial application.

From an industrial perspective, the enzymatic hydrolysis of blue crab by-products presents promising opportunities for large-scale application. The use of commercial proteases such as Alcalase and Protamex is particularly advantageous, as these enzymes are already widely employed in the food and biotechnology sectors, ensuring technical feasibility for scale-up [12,85]. Although enzyme costs may initially represent a constraint, the superior efficiency of Alcalase observed in this study could offset production expenses by maximizing hydrolysis yields and bioactive peptide recovery [12,64].

It is important to note that blue crabs are benthic organisms and may accumulate environmental contaminants, including heavy metals such as lead (Pb), cadmium (Cd), mercury (Hg), and arsenic (As), which could pose risks to human and animal health. [9,10]. Although BCBP hydrolysates demonstrated promising antioxidant and bioactive properties, their heavy metal content was not assessed in this study. Addressing these safety aspects is essential before industrial application in the food, nutraceutical, and feed sectors. Future studies should focus on evaluating heavy metal levels and other possible contaminants to confirm both safety and efficacy, thereby enabling the sustainable valorization of blue crab by-products for industrial uses while supporting circular economy principles.

Moreover, valorizing BCBP through enzymatic hydrolysis aligns strongly with circular economy and bioeconomy principles [15,78]. This approach reduces the environmental burden associated with marine waste management while generating value-added products with applications across food, feed, nutraceutical, and pharmaceutical sectors [13,78,86]. The integration of hydrolysis technologies into existing seafood processing chains could therefore contribute to improving resource efficiency, reducing reliance on unsustainable protein sources, and promoting the long-term sustainability and competitiveness of the fishery industry [12,84,85].

## 4. Conclusions

This study demonstrates that blue crab by-products, currently considered as waste, can be efficiently converted into valuable protein hydrolysates through enzymatic hydrolysis. The obtained hydrolysates showed a high degree of hydrolysis (DH% > 10%), confirming their suitability for producing bioactive peptides. Importantly, they exhibited strong antioxidant activity, both in chemical assays and in protecting human fibroblasts (HS-68) from H_2_O_2_-induced oxidative stress.

These findings open opportunities for multiple applications, transforming an environmental challenge into an economic resource. Hydrolysates can serve as functional ingredients in various sectors, such as the food industry, where they can be used as natural additives to extend the shelf life of perishable products. They can also be used in aquaculture as an alternative, functional protein source in feed to promote fish health and growth.

Their production is potentially scalable, and the valorization of processing waste could provide economic benefits and promote environmental sustainability. Ultimately, the valorization of blue crab by-products reduces its environmental impact, creates economic value, and exemplifies the principles of the circular economy, providing a sustainable and profitable model for the local fishing industry.

## Figures and Tables

**Table 1:** Yield and waste (mean ± SD) (%) of blue crabs obtained from the fish processing industry.

	%
Yield meat	21.87 ± 2.38
Waste	72.45 ± 4.08

**Table 2:** Proximate composition (dw) (mean ± SD; *n* = 3) of blue crab by-products BCBP.

Components	%
Ash	35.38 ± 0.30
Total Lipid	2.57 ± 0.09
Protein	31.21 ± 1.12
Chitin	10.57 ± 0.66

## Data Availability

The data presented in this study are available on request from the corresponding authors.

## Abbreviations

The following abbreviations are used in this manuscript:

BCBP	Blue crab by-products
SDS PAGE	Sodium Dodecyl Sulphate Polyacrylamide Gel Electrophoresis
BSA	Bovine Serum Albumin
DPPH	2,2-diphenyl-1-picrylhydrazyl
ABTS	2′-azinobis-(3-ethylbenzothiazoline-6-sulfonic acid)
FRAP	Ferric-Reducing Antioxidant Power
HS68	Human skin fibroblasts
MTT	3-(4,5-dimethyl-2-yl)-2,5-diphenyltetrazolium bromide

## References

[1] Kevrekidis K., Kevrekidis T., Mogias A., Boubonari T., Kantaridou F., Kaisari N., Malea P., Dounas C., Thessalou-Legaki M. (2023). Fisheries Biology and Basic Life-Cycle Characteristics of the Invasive Blue Crab *Callinectes sapidus* Rathbun in the Estuarine Area of the Evros River (Northeast Aegean Sea, Eastern Mediterranean). J. Mar. Sci. Eng..

[2] Nehring S. (2011). Invasion History and Success of the American Blue Crab *Callinectes sapidus* in European and Adjacent Waters. In the Wrong Place—Alien Marine Crustaceans: Distribution, Biology and Impacts.

[3] Mancinelli G., Bardelli R., Zenetos A. (2021). A global occurrence database of the Atlantic blue crab *Callinectes sapidus*. Sci. Data.

[4] Khamassi F., Bahri W.R., Bhouri A.M., Chaffai A., Soufikechaou E., Ghanem R., Souissi J. (2022). Ben Biochemical composition, nutritional value, and socio-economic impacts of the invasive crab *Callinectes sapidus* Rathbun, 1896 in central Mediterranean Sea. Mediterr. Mar. Sci..

[5] Taybi A.F., Mabrouki Y. (2020). The American Blue Crab *Callinectes sapidus* Rathbun, 1896 (Crustacea: Decapoda: Portunidae) is Rapidly Expanding Through the Mediterranean Coast of Morocco. Thalassas.

[6] Marchessaux G., Mangano M.C., Bizzarri S., M’Rabet C., Principato E., Lago N., Veyssiere D., Garrido M., Scyphers S.B., Sarà G. (2023). Invasive blue crabs and small-scale fisheries in the Mediterranean sea: Local ecological knowledge, impacts and future management. Mar. Policy.

[7] Arena R., Renda G., Ottaviani Aalmo G., Debeaufort F., Maria Messina C., Santulli A. (2024). Valorization of the Invasive Blue Crabs (*Callinectes sapidus*) in the Mediterranean: Nutritional Value, Bioactive Compounds and Sustainable By-Products Utilization. Mar. Drugs.

[8] Su W., Yu S., Wu D., Xia M., Wen Z., Yao Z., Tang J., Wu W. (2019). A critical review of cast-off crab shell recycling from the perspective of functional and versatile biomaterials. Environ. Sci. Pollut. Res..

[9] Nekvapil F., Ganea I.V., Ciorîță A., Hirian R., Ogresta L., Glamuzina B., Roba C., Pinzaru S.C. (2021). Wasted biomaterials from crustaceans as a compliant natural product regarding microbiological, antibacterial properties and heavy metal content for reuse in blue bioeconomy: A preliminary study. Materials.

[10] Perelló E., Pinya S., Box A., Sureda A., Compa M. (2025). Assessing heavy metal accumulation in the invasive blue crab (*Callinectes sapidus*): Environmental and human health implications. Environ. Sci. Pollut. Res..

[11] Espinales C., Romero-Peña M., Calderón G., Vergara K., Cáceres P.J., Castillo P. (2023). Collagen, protein hydrolysates and chitin from by-products of fish and shellfish: An overview. Heliyon.

[12] Antunes-Valcareggi S.A., Ferreira S.R.S., Hense H. (2017). Enzymatic hydrolysis of blue crab (*Callinectes sapidus*) waste processing to obtain chitin, protein, and astaxanthin-enriched extract. Int. J. Environ. Agric. Res..

[13] Félix-Valenzuela L., Higuera-Ciapara I., Goycoolea-Valencia F., Argüelles-Monal W. (2001). Supercritical CO_2_/ethanol extraction of astaxanthin from blue crab (*Callinectes sapidus*) shell waste. J. Food Process Eng..

[14] Harnedy P.A., Fitzgerald R.J. (2011). Bioactive proteins, peptides, and amino acids from macroalgae. J. Phycol..

[15] Messina C.M., Manuguerra S., Arena R., Renda G., Ficano G., Randazzo M., Fricano S., Sadok S., Santulli A. (2021). In Vitro Bioactivity of Astaxanthin and Peptides from Hydrolisates of Shrimp (*Parapenaeus longirostris*) By-Products: From the Extraction Process to Biological Effect Evaluation, as Pilot Actions for the Strategy “From Waste to Profit”. Mar. Drugs.

[16] Caruso G., Floris R., Serangeli C., Di Paola L. (2020). Fishery Wastes as a Yet Undiscovered Treasure from the Sea: Biomolecules Sources, Extraction Methods and Valorization. Mar. Drugs.

[17] Coppola D., Lauritano C., Esposito F.P., Riccio G., Rizzo C., De Pascale D., Santulli A. (2021). Fish Waste: From Problem to Valuable Resource. Mar. Drugs.

[18] Venugopal V. (2022). Green processing of seafood waste biomass towards blue economy. Curr. Res. Environ. Sustain..

[19] Thirukumaran R., Anu Priya V.K., Krishnamoorthy S., Ramakrishnan P., Moses J.A., Anandharamakrishnan C. (2022). Resource recovery from fish waste: Prospects and the usage of intensified extraction technologies. Chemosphere.

[20] Hajji S., Kchaou H., Bkhairia I., Ben Slama-Ben Salem R., Boufi S., Debeaufort F., Nasri M. (2021). Conception of active food packaging films based on crab chitosan and gelatin enriched with crustacean protein hydrolysates with improved functional and biological properties. Food Hydrocoll..

[21] Debeaufort F. (2021). Active biopackaging produced from by-products and waste from food and marine industries. FEBS Open Bio.

[22] Jimenez-Champi D., Romero-Orejon F.L., Muñoz A.M., Ramos-Escudero F. (2024). The Revalorization of Fishery By-Products: Types, Bioactive Compounds, and Food Applications. Int. J. Food Sci..

[23] Yomar-Hattori G., Sampaio-Sant’Anna B., Amaro-Pinheiro M.A. (2006). Meat yield of *Callinectes bocourti* A. Milne Edwards, 1879 (Crustacea, Portunidae) in Iguape, São Paulo, Brazil. Investig. Mar..

[24] AOAC (1990). Official Methods of Analysis: Changes in Official Methods of Analysis Made at the Annual Meeting.

[25] Folch J., Lees M., Stanley G.H.S. (1957). A simple method for the isolation and purification of total lipids from animal tissues. J. Biol. Chem..

[26] Association of Official Analytical Chemists (1992). Official Method, 981.10 Crude protein in meat block digestion method. J. AOAC Int..

[27] Khiari Z., Kelloway S., Mason B. (2020). Turning Invasive Green Crab (*Carcinus maenas*) into Opportunity: Recovery of Chitin and Protein Isolate Through Isoelectric Solubilization/Precipitation. Waste Biomass Valorization.

[28] Sajomsang W., Gonil P. (2010). Preparation and characterization of α-chitin from cicada sloughs. Mater. Sci. Eng. C.

[29] Rutledge J.E. (1971). Decalcification of Crustacean Meals. J. Agric. Food Chem..

[30] Bolat Y., Bilgin Ş., Günlü A., Izci L., Koca S.B., Çetinkaya S., Koca H.U. (2010). Chitin-chitosan yield of freshwater crab (*Potamon potamios*, Olivier 1804) shell. Pak. Vet. J..

[31] Messina C., Renda G., Randazzo M., Laudicella A., Gharbi S., Pizzo F., Morghese M., Santulli A. (2015). Extraction of bioactive compounds from shrimp waste. Bull. Inst. Natl. Sci. Technol. Mer.

[32] Dumay J., Donnay-Moreno C., Barnathan G., Jaouen P., Bergé J.P. (2006). Improvement of lipid and phospholipid recoveries from sardine (*Sardina pilchardus*) viscera using industrial proteases. Process Biochem..

[33] De Holanda H.D., Netto F.M. (2006). Recovery of components from shrimp (*Xiphopenaeus kroyeri*) processing waste by enzymatic hydrolysis. J. Food Sci..

[34] Lowry O.H., Rosebrough N.J., Farr A.L., Randall R.J. (1951). Protein measurement with the Folin phenol reagent. J. Biol. Chem..

[35] Re R., Pellegrini N., Proteggente A., Pannala A., Yang M., Rice-Evans C. (1999). Antioxidant activity applying an improved ABTS radical cation decolorization assay. Free Radic. Biol. Med..

[36] Lanzoni D., Skřivanová E., Rebucci R., Crotti A., Baldi A., Marchetti L., Giromini C. (2023). Total Phenolic Content and Antioxidant Activity of In Vitro Digested Hemp-Based Products. Foods.

[37] Abdelaleem M.A., Elbassiony K.R.A. (2021). Evaluation of phytochemicals and antioxidant activity of gamma irradiated quinoa (*Chenopodium quinoa*). Braz. J. Biol..

[38] Messina C.M., Troia A., Arena R., Manuguerra S., Ioannou T., Curcuraci E., Renda G., Hellio C., Santulli A. (2019). Species-specific antioxidant power and bioactive properties of the extracts obtained from wild mediterranean *Calendula* spp. (Asteraceae). Appl. Sci..

[39] Manuguerra S., Caccamo L., Mancuso M., Arena R., Rappazzo A.C., Genovese L., Santulli A., Messina C.M., Maricchiolo G. (2020). The antioxidant power of horseradish, *Armoracia rusticana*, underlies antimicrobial and antiradical effects, exerted in vitro. Nat. Prod. Res..

[40] Mosmann T. (1983). Rapid colorimetric assay for cellular growth and survival: Application to proliferation and cytotoxicity assays. J. Immunol. Methods.

[41] Messina C.M., Manuguerra S., Renda G., Santulli A. (2019). Biotechnological Applications for the Sustainable Use of Marine By-products: In Vitro Antioxidant and Pro-apoptotic Effects of Astaxanthin Extracted with Supercritical CO_2_ from *Parapeneus longirostris*. Mar. Biotechnol..

[42] Messina C.M., Pizzo F., Santulli A., Bušelić I., Boban M., Orhanović S., Mladineo I. (2016). *Anisakis pegreffii* (Nematoda: Anisakidae) products modulate oxidative stress and apoptosis-related biomarkers in human cell lines. Parasites Vectors.

[43] Abbes M., Baati H., Guermazi S., Messina C., Santulli A., Gharsallah N., Ammar E. (2013). Biological properties of carotenoids extracted from *Halobacterium halobium* isolated from a Tunisian solar saltern. BMC Complement. Altern. Med..

[44] Zou Y., Robbens J., Heyndrickx M., Debode J., Raes K. (2021). Bioprocessing of marine crustacean side-streams into bioactives: A review. J. Chem. Technol. Biotechnol..

[45] Martínez M.A., Velazquez G., Cando D., Núñez-Flores R., Borderías A.J., Moreno H.M. (2017). Effects of high pressure processing on protein fractions of blue crab (*Callinectes sapidus*) meat. Innov. Food Sci. Emerg. Technol..

[46] Cretton M., Malanga G., Mazzuca Sobczuk T., Mazzuca M. (2021). Lipid Fraction from Industrial Crustacean Waste and Its Potential as a Supplement for the Feed Industry: A Case Study in Argentine Patagonia. Waste Biomass Valorization.

[47] Naczk M., Williams J., Brennan K., Liyanapathirana C., Shahidi F. (2004). Compositional characteristics of green crab (*Carcinus maenas*). Food Chem..

[48] Sudhakar M., Manivannan K., Soundrapandian P. (2009). Nutritive Value of Hard and Soft Shell Crabs of *Portunus sanguinolentus* (Herbst). Int. J. Anim. Vet. Adv..

[49] Pinheiro A.C.D.A.S., Martí-Quijal F.J., Barba F.J., Tappi S., Rocculi P. (2021). Innovative non-thermal technologies for recovery and valorization of value-added products from crustacean processing by-products—An opportunity for a circular economy approach. Foods.

[50] Ahmadkelayeh S., Hawboldt K. (2020). Extraction of lipids and astaxanthin from crustacean by-products: A review on supercritical CO_2_ extraction. Trends Food Sci. Technol..

[51] Parthiban F., Balasundari S., Gopalakannan A., Rathnakumar K., Felix S. (2017). Comparison of the Quality of Chitin and Chitosan from Shrimp, Crab and Squilla Waste. Curr. World Environ..

[52] Vilasoa-Martínez M., López-Hernández J., Lage-Yusty M.A. (2007). Protein and amino acid contents in the crab, *Chionoecetes opilio*. Food Chem..

[53] Jabeur F., Mechri S., Mensi F., Gharbi I., Naser Y.B., Kriaa M., Bejaoui N., Bachouche S., Badis A., Annane R. (2022). Extraction and characterization of chitin, chitosan, and protein hydrolysate from the invasive Pacific blue crab, *Portunus segnis* (Forskål, 1775) having potential biological activities. Environ. Sci. Pollut. Res..

[54] Giordano D., Costantini M., Coppola D., Lauritano C., Núñez Pons L., Ruocco N., di Prisco G., Ianora A., Verde C. (2018). Biotechnological Applications of Bioactive Peptides From Marine Sources. Adv. Microb. Physiol..

[55] Mittal A., Singh A., Xavier M., Ravishankar C.N., Benjakul S. (2024). Chitin, Chitosan, and their Derivatives from Seafood Waste and Processing Byproducts. Fish Waste to Valuable Products.

[56] Jabeen F., Younis T., Sidra S., Muneer B., Nasreen Z., Saleh F., Mumtaz S., Saeed R.F., Abbas A.S. (2021). Extraction of chitin from edible crab shells of *Callinectes sapidus* and comparison with market purchased chitin. Braz. J. Biol..

[57] Metin C., Alparslan Y., Baygar T., Baygar T. (2019). Physicochemical, Microstructural and Thermal Characterization of Chitosan from Blue Crab Shell Waste and Its Bioactivity Characteristics. J. Polym. Environ..

[58] Kaya M., Dudakli F., Asan-Ozusaglam M., Cakmak Y.S., Baran T., Mentes A., Erdogan S. (2016). Porous and nanofiber α-chitosan obtained from blue crab (*Callinectes sapidus*) tested for antimicrobial and antioxidant activities. LWT.

[59] Pires C., Marques A., Carvalho M.L., Batista I.B. (2017). Chemical Characterization of *Cancer pagurus*, *Maja squinado*, *Necora puber* and *Carcinus maenas* shells. Poult. Fish. Wildl. Sci..

[60] Cahú T.B., Santos S.D., Mendes A., Córdula C.R., Chavante S.F., Carvalho L.B., Nader H.B., Bezerra R.S. (2012). Recovery of protein, chitin, carotenoids and glycosaminoglycans from Pacific white shrimp (*Litopenaeus vannamei*) processing waste. Process Biochem..

[61] Messina C.M., Arena R., Manuguerra S., Pericot Y., Curcuraci E., Kerninon F., Renda G., Hellio C., Santulli A. (2021). Antioxidant Bioactivity of Extracts from Beach Cast Leaves of *Posidonia oceanica* (L.) Delile. Mar. Drugs.

[62] Camargo T.R., Mantoan P., Ramos P., Monserrat J.M., Prentice C., Fernandes C.C., Zambuzzi W.F., Valenti W.C. (2021). Bioactivity of the Protein Hydrolysates Obtained from the Most Abundant Crustacean Bycatch. Mar. Biotechnol..

[63] Dey S.S., Dora K.C. (2014). Optimization of the production of shrimp waste protein hydrolysate using microbial proteases adopting response surface methodology. J. Food Sci. Technol..

[64] Gunasekaran J., Kannuchamy N., Kannaiyan S., Chakraborti R., Gudipati V. (2015). Protein Hydrolysates from Shrimp (*Metapenaeus dobsoni*) Head Waste: Optimization of Extraction Conditions by Response Surface Methodology. J. Aquat. Food Prod. Technol..

[65] Shaibani M.E., Heidari B., Khodabandeh S., Shahangian S., Mirdamadi S., Mirzaei M. (2020). Antioxidant and antibacterial properties of protein hydrolysate from rocky shore crab, *Grapsus albolineathus*, as affected by progress of hydrolysis. Int. J. Aquat. Biol..

[66] Ovissipour M., Abedian A., Motamedzadegan A., Rasco B., Safari R., Shahiri H. (2009). The effect of enzymatic hydrolysis time and temperature on the properties of protein hydrolysates from Persian sturgeon (*Acipenser persicus*) viscera. Food Chem..

[67] Zamora-Sillero J., Ramos P., Monserrat J.M., Prentice C. (2017). Evaluation of the Antioxidant Activity In Vitro and in Hippocampal HT-22 Cells System of Protein Hydrolysates of Common Carp (*Cyprinus carpio*) By-Product. J. Aquat. Food Prod. Technol..

[68] Das A., Nayak Y., Dash S. (2021). Fish protein hydrolysate production, treatment methods and current potential uses: A review. Int. J. Fish. Aquat. Stud..

[69] Latorres J.M., Rios D.G., Saggiomo G., Wasielesky W., Prentice-Hernandez C. (2018). Functional and antioxidant properties of protein hydrolysates obtained from white shrimp (*Litopenaeus vannamei*). J. Food Sci. Technol..

[70] Bordbar S., Ebrahimpour A., Zarei M., Hamid A.A., Saari N. (2018). Alcalase-generated proteolysates of stone fish (*Actinopyga lecanora*) flesh as a new source of antioxidant peptides. Int. J. Food Prop..

[71] Jiang W., Hu S., Li S., Liu Y. (2017). Biochemical and antioxidant properties of peptidic fraction generated from crab (*Portunus trituberculatus*) shells by enzymatic hydrolysis. Int. J. Food Sci. Technol..

[72] Kim S.B., Yoon N.Y., Shim K.B., Lim C.W. (2016). Antioxidant and angiotensin I-converting enzyme inhibitory activities of northern shrimp (*Pandalus borealis*) by-products hydrolysate by enzymatic hydrolysis. Fish. Aquat. Sci..

[73] Aklakur M. (2018). Natural antioxidants from sea: A potential industrial perspective in aquafeed formulation. Rev. Aquac..

[74] Di Bernardini R., Harnedy P., Bolton D., Kerry J., O’Neill E., Mullen A.M., Hayes M. (2011). Antioxidant and antimicrobial peptidic hydrolysates from muscle protein sources and by-products. Food Chem..

[75] Nikoo M., Regenstein J.M., Yasemi M. (2023). Protein Hydrolysates from Fishery Processing By-Products: Production, Characteristics, Food Applications, and Challenges. Foods.

[76] Nikoo M., Benjakul S., Yasemi M., Ahmadi Gavlighi H., Xu X. (2019). Hydrolysates from rainbow trout (*Oncorhynchus mykiss*) processing by-product with different pretreatments: Antioxidant activity and their effect on lipid and protein oxidation of raw fish emulsion. LWT.

[77] Schaich K.M., Tian X., Xie J. (2015). Reprint of “Hurdles and pitfalls in measuring antioxidant efficacy: A critical evaluation of ABTS, DPPH, and ORAC assays”. J. Funct. Foods.

[78] Martínez-Montaño E., Sarmiento-Machado R.M., Osuna-Ruíz I., Benítez-García I., Pacheco-Aguilar R., Navarro-Peraza R.S., Sánchez M.E.L., Ortiz A.V., Báez L.J.G., Bañuelos-Vargas I. (2022). Effect of Degree of Hydrolysis on Biochemical Properties and Biological Activities (Antioxidant and Antihypertensive) of Protein Hydrolysates from Pacific Thread Herring (*Ophistonema libertate*) Stickwater. Waste Biomass Valorization.

[79] Zamora-Sillero J., Gharsallaoui A., Prentice C. (2018). Peptides from Fish By-product Protein Hydrolysates and Its Functional Properties: An Overview. Mar. Biotechnol..

[80] Guerard F., Sumaya-Martinez M.T., Laroque D., Chabeaud A., Dufossé L. (2007). Optimization of free radical scavenging activity by response surface methodology in the hydrolysis of shrimp processing discards. Process Biochem..

[81] Harnedy P.A., FitzGerald R.J. (2012). Bioactive peptides from marine processing waste and shellfish: A review. J. Funct. Foods.

[82] Yoon N.Y., Shim K.B., Lim C.W., Kim S.B. (2013). Antioxidant and angiotensin I converting enzyme inhibitory activities of red snow crab *Chionoecetes japonicas* shell hydrolysate by enzymatic hydrolysis. Fish. Aquat. Sci..

[83] Wong F., Ng W., Ooi A., Lem F., Chai T. (2025). Carp-Derived Antioxidant Peptides and Hydrolysates: Biological Effects and Potential Applications in Health and Food. Antioxidants.

[84] Sun K.L., Gao M., Wang Y.Z., Li X.R., Wang P., Wang B. (2022). Antioxidant Peptides From Protein Hydrolysate of Marine Red Algae *Eucheuma cottonii*: Preparation, Identification, and Cytoprotective Mechanisms on H_2_O_2_ Oxidative Damaged HUVECs. Front. Microbiol..

[85] Wang B., Chi C.F. (2025). Marine Bioactive Peptides—Structure, Function, and Application 2.0. Mar. Drugs.

[86] Xu F., Zhang Y., Qiu Y., Yang F., Liu G., Dong X., Chen G., Cao C., Zhang Q., Zhang S. (2022). Three novel antioxidant peptides isolated from C-phycocyanin against H_2_O_2_-induced oxidative stress in zebrafish via Nrf2 signaling pathway. Front. Mar. Sci..

[87] Li Q., Liu Z., Yang G., Zhang D., Qin H., Xia B., Liu S., Chen J. (2025). Supplementation of Enzymatic Hydrolysate in Low-Fishmeal and Low-Crop Diet Improves Growth, Antioxidant Capacity, and Immunity of Juvenile Sea Cucumber *Apostichopus japonicus* (Selenka). Fishes.

